# Comprehensive phytochemical characterization of *Persea americana* Mill. fruit via UPLC/HR-ESI–MS/MS and anti-arthritic evaluation using adjuvant-induced arthritis model

**DOI:** 10.1007/s10787-023-01365-z

**Published:** 2023-11-07

**Authors:** Dina Atef Waly, Aisha Hussein Abou Zeid, Hanan Naeim Attia, Kawkab A. Ahmed, El-Sayeda Ahmed El-Kashoury, Ali Mahmoud El Halawany, Reda Sayed Mohammed

**Affiliations:** 1 Department of Pharmacognosy, National Research Centre (ID: 60014618), 33-Elbohouth St (Former El-Tahrir St.), Dokki, P.O. 12622, Giza, Egypt; 2 Medicinal and Pharmaceutical Chemistry Department (Pharmacology Group), National Research Centre (ID: 60014618), 33-Elbohouth St (Former El-Tahrir St.), Dokki, P.O. 12622, Giza, Egypt; 3 Department of Pathology, Faculty of Veterinary Medicine, Cairo University, P.O. 12211 Giza, Egypt; 4 Pharmacognosy Department, Faculty of Pharmacy, Cairo University, Kasr-El Ainy Street, Cairo, 11562 Egypt

**Keywords:** *Persea americana*, DPPH, IL-6, TNF-α, Arthritis, Complete Freund’s adjuvant (CFA), Lyso-glycerophospholipids, Lyso-glycerophosphoethanolamines, Lyso-glycerophosphocholines, UPLC/HR-ESI–MS/MS

## Abstract

*Persea americana* Mill. (avocado fruit) has many health benefits when added to our diet due to various pharmacological activities, such as preventing bone loss and inflammation, modulating immune response and acting as an antioxidant. In the current study, the total ethanol extract (TEE) of the fruit was investigated for in vitro antioxidant and anti-inflammatory activity via DPPH and cyclooxygenase enzyme inhibition. Biological evaluation of the antiarthritic effect of the fruit extract was further investigated in vivo using Complete Freund’s Adjuvant (CFA) arthritis model, where the average percentages of body weight change, inhibition of paw edema, basal paw diameter/weight and spleen index were estimated for all animal groups. Inflammatory mediators such as serum IL-6 and TNF-α were also determined, in addition to histopathological examination of the dissected limbs isolated from all experimental animals. Eighty-one metabolites belonging to different chemical classes were detected in the TEE of *P. americana* fruit via UPLC/HR-ESI–MS/MS. Two classes of lyso-glycerophospholipids; lyso-glycerophosphoethanolamines and lysoglycerophosphocholines were detected for the first time in avocado fruit in the positive mode. The TEE of fruit exhibited significant antioxidant and anti-inflammatory activity in vitro. In vivo anti-arthritic activity of the fruit TEE improved paw parameters, inflammatory mediators and spleen index. Histopathological findings showed marked improvements in the arthritic condition of the excised limbs. Therefore, avocado fruit could be proposed to be a powerful antioxidant and antiarthritic natural product.

## Introduction

Rheumatoid arthritis (RA) is an autoimmune disorder characterized by systemic inflammation that damages joints, impairing mobility and perhaps decreasing lifespan (Smolen and Aletaha 2015). According to Prescha et al. (2018), the clinical condition of RA was significantly correlated with the availability of foods and nutrients that regulate antioxidant and anti-inflammatory defense mechanisms. Food quality assessment and its relationship to anti-inflammatory status in RA patients may help to discover dietary practices and nutrient intake that are crucial for enhancing antioxidant defenses, thus delaying RA symptoms (Jalili et al. 2014). Due to its high polyphenolic flavonoid content, which has been associated with antioxidant, anti-inflammatory and analgesic properties, adding fruit like avocado to RA patients’ diet may help to reduce inflammatory symptoms. Alligator pear (*Persea Americana* Mill., Lauraceae), is an edible fruit with creamy smooth flesh and bumpy skin, (Bhuyan et al. 2019). It is a very nutritious fruit as it contains fat-soluble vitamins which are less common in other fruits and valuable constituents including A, C, E, K1, folate, B-6, niacin, pantothenic acid, riboflavin choline, carotenoids, phytosterols and high-monounsaturated fatty acids (Dreher and Davenport 2013). The seeds and peels are rich sources of bioactive metabolites, such as phytosterols, triterpenes, catechins, hydroxycinnamic acids, pro-anthocyanidins, and glycosylated abscisic acid derivatives. The bark, fruit and leaf are used in traditional medicine in South and Central America. They are used for the treatment of various ailments such as menorrhagia, hypertension, stomach problems, bronchitis, diarrhea and diabetes (Adeyemi et al. 2002). Several health benefits were reported including antioxidant actions (Rodríguez-Carpena et al. 2011). The aqueous leaf extract of avocado produced a significant inhibition of carrageenan-induced swelling with a similar effect to that produced by indomethacin in the same duration. (Adeyemi et al. 2002). Natural products and their constituents possess advantages over synthetic alternatives with respect to potency, accessibility, low cost, lower side effects, superior safety and efficacy. These therapeutic natural phytoconstituents include steroids, glycosides, phenolics, flavonoids, fatty acids that have anti-inflammatory properties in addition to their ability to halt inflammatory processes (Yatoo et al. 2018).

## Aim of the current study

The main aim of the study is to perform a complete phytochemical characterization of Avocado fruit extract, investigate the in vitro antioxidant, anti-inflammatory and in vivo antiarthritic activity, in an attempt to use the fruit as a protective and curative natural plant material for arthritis. Possible beneficial effect of two doses of fruit extract was compared to methotrexate, which belongs to a class known as disease-modifying antirheumatic drugs (DMARDs) and is considered the first line of treatment for rheumatoid arthritis but possesses many adverse events reported in the literature. The effect of avocado consumption on CFA-induced arthritis in rats was studied in addition to histopathological examination of excised hindlimb.

## Material and methods

### Plant material

The fruits of avocado (*Persea americana* Mill., Lauraceae) were collected during May–June 2017 from local markets and were kindly identified by the Fruit Department, Institute of Agriculture, the National Research Centre.

### Chemicals and reagents

Antioxidants reagents: 1,1-diphenyl-2-picryl-hydroxyl (DPPH) and Ascorbic acid were purchased from Sigma-Aldrich (Darmstadt, Germany). In vitro anti-inflammatory activity was assessed using a kit provided by Cayman Chemical Company (USA). Kits for estimation of rat tumor necrosis factor alpha (TNF-α) and interleukin-6 (IL-6) were purchased from Elabscience (USA)***.*** Indomethacin, Celecoxib and methotrexate were obtained from Alkahira Company (Egypt), Sigma-Aldrich (USA) and Shanxi PUDE (Pharmaceutical Co, Ltd. imported by Techno Pharma, China), respectively. Ethanol was purchased from Adwic (Cairo, Egypt). CFA vial (heat-killed dried *Mycobacterium butyricum* (10 mg) in 1.5 ml mannide monooleate and 8.5 ml paraffin oil) was obtained from Sigma (USA).

### Preparation of the extract

One kilogram of the deseeded fruits was extracted with 80% ethanol till exhaustion. The combined filtered extract was evaporated to dryness under vacuum to yield 80 g of total ethanol extract (TEE).

### In vitro study

#### Evaluation of radical scavenging potential

DPPH radical scavenging activity of TEE of avocado fruit samples at 62.5, 125, 250, 500, and 1000 µg/ml concentrations was assessed according to the method adopted by Ibrahim et al. (2021). The absorbance was measured spectrophotometrically at 517 nm against ascorbic acid as the reference standard. The % inhibition of DPPH was calculated as follows:

$$\% Inhibition =\left[\frac{{A}_{c}-{A}_{t}}{{A}_{c}}\right]\times 100,$$

Where A_c_ = absorbance of the control, A_t_ = the absorbance of the sample.

#### Evaluation of anti-inflammatory activity

The cyclooxygenase inhibition efficacy of TEE of avocado fruit was performed. Procedures were followed as per manufacturer's instructions (Blobaum and Marnett 2007). Indomethacin and celecoxib were used as standard anti-inflammatory compounds against COX1 and COX2 activity.

### In vivo study

#### Animals

Adult Sprague Dawley rats weighing 180–200 g were purchased from the animal breeding colony of National Research Center (Giza, Egypt). They were housed and kept under standardized laboratory conditions (room temperature adjusted to 23 ± 2 °C, relative humidity of 55 ± 5% and a 12-h light/dark exposure with proper ventillation) throughout the study period (28 days), with acclimatization for one week prior to experimentation. Rats were fed standard pellet diet with free access to water. All procedures were in accordance with regulations by the National Research Centre Ethics Committee (Approval Number: 16474).

### Acute toxicity study

The acute toxicity test for TEE of avocado fruit was carried out to evaluate any possible toxicological signs using 'The Limit Test' with a subjected dose of 2 and 5 g/kg orally according to Economic Cooperation and Development (OECD) 425 guidelines (OECD 2008).

### Anti-arthritic activity

#### Complete Freund’s adjuvant (CFA) arthritis

CFA arthritic model is sensitive to anti-inflammatory and immune-inhibiting medicines for the study of pathophysiological and pharmacological control of the inflammatory process (Bevaart et al. 2010). Arthritis was induced by intraplantar injection of 0.1 ml of adjuvant into the surface of right hind paw of rats (Lao et al. 2001). The left hind paw was injected with an equivalent volume of saline (Noh et al. 2021).

#### Experimental design

Rats were randomly assigned to five groups (n = 8) and received their respective treatments as follows: Group I: served as the negative control group which received oral and intraplanar injections of normal saline. Group II served as positive control, receiving an intraplanar injection of 0.1 ml CFA. Group III: received CFA followed by daily oral methotrexate (MTX 0.25 mg/kg) for 28 consecutive days as the reference standard drug. Groups IV and V received CFA followed by a daily oral dose of 500 and 1000 (mg/kg) of TEE of avocado fruit for 28 consecutive days, respectively according to results observed from the acute toxicity study.

#### Collection of data

Paw edema and joint thickness were monitored on the 7th, 14th, 21st, and 28th days by using a plethysmometer and vernier calipers, respectively to confirm the development of arthritis. The mean percent change of injected paw diameter with respect to basal values and percent inhibition of paw edema was calculated at the end of the study period using the following formula: $\% Inhibtion\, in\, paw\, edema = 100\times \left(1-\frac{VT}{VC}\right)$

Where VC = mean paw edema volume in the control group and VT = mean paw edema volume in the drug-treated group.

At the end of the experimental period (28 days), animals were euthanized and sacrificed by decapitation. Blood was collected and centrifuged at a speed of 4000 rpm at 4 °C for separation of serum which was deep frozen at (− 80 °C) till the time of biochemical estimations. Both left and right hind paws were excised then weighed. for determination of mean % paw weight of the basal value of the right hind paw. Finally, hindlimbs from each group were immersed in buffered formalin (10%) for histopathological investigations.

The relative organ weight of the spleen is indicative of immunological function. Therefore, the spleen of all animals was isolated, washed with cold saline then blotted dry between two filter papers and weighed to estimate spleen index % calculated as follows:

$$\mathrm{Spleen\, index \% }=\frac{weight \,of\, spleen}{Final\, body\, weight} \times 100$$

### Biochemical analysis

TNF-α and IL-6 were estimated by using a solid-phase sandwich ELISA test kit for rats, according to the manufacturer's instructions (Elabscience, USA, Elabscience Biotechnology Inc, USA).

### Statistical analysis

Data collected from the animal study were analyzed using GraphPad Prism software (San Diego, CA, USA) by performing ANOVA (1-way) followed by Tukey’s post hoc analysis with a probability level set at *p* < 0.05 for significance.

### Histopathological studies

Dissected hind limbs were decalcified in 10% formic acid for 14 days. Samples were dehydrated in ethanol series immersion, cleared in xylene, and embedded in paraffin. After dehydration in graded alcohol and clearing in xylene, tissue blocks were impregnated and embedded in paraffin. For histopathological examination, 5 µm-thick sections were stained with hematoxylin and eosin (H&E) (Mepham 1991).

### Phytochemical study

#### UPLC/HR-ESI–MS/MS metabolomic profiling of TEE of avocado fruit

##### Sample preparation

Samples for HPLC analysis were prepared by adding 1 ml of the mobile phase working solution MP-WS (acetonitrile:methanol:water, 25:25:50) to 50 mg of TEE of avocado, then the sample was prepared as mentioned by Ragheb et al. (2023) Sample (25 μl) was injected on positive and negative modes, additionally 25 μl of MP-WS was injected as a blank sample.

##### LC–MS/MS analysis

LC–MS/MS measurements in both ionization modes (+ve and −ve) of avocado extract were carried out on Exion LC High flow LC (Sciex hardware) coupled with triple TOF 5600 + IDA acquisition for LC-QTOF control according to Farid et al. (2022). Each injected sample (10 μl) was passed through a Phenomenex In-Line filter disk (3.0 mm, 0.5 μm). Waters X select HSS T3 (2.1 × 150 mm, 2.5 μm) column is used, temperature (40 °C). The mobile phase A containing 5 mM NH_4_HCO_2_ buffer at pH 3 with 1% methanol was used for the +ve ionization mode while 5 mM NH_4_ HCO_2_ buffer at pH 8 containing 1% methanol for the −ve mode. Acetonitrile100% was used as a mobile phase B for both ion modes. The flow rate was 0.3 ml/min, a linear gradient for a run duration for 28 min was used: (90% A and 10% B) for 20 min, (10% A and 90% B) for 5 min then (90% A and 10% B) for the remaining 3 min. For both ion modes, a capillary voltage of 4500 V, 500 °C is a source temperature, and the MS acquisition is ranged from 50 to 1000 *m/z*. Further, the peaks and spectra were processed using the Analyst TF 1.7.1 and PeakView^®^ 1.2 Softwares (SCIEX, Framingham, MA, USA). The tentative identification of the compounds was carried out by comparison of their masses, fragmentation pattern, and molecular formulae with those in the literature.

## Results and discussion

### In vitro antioxidant activity

**Fig. 1:**
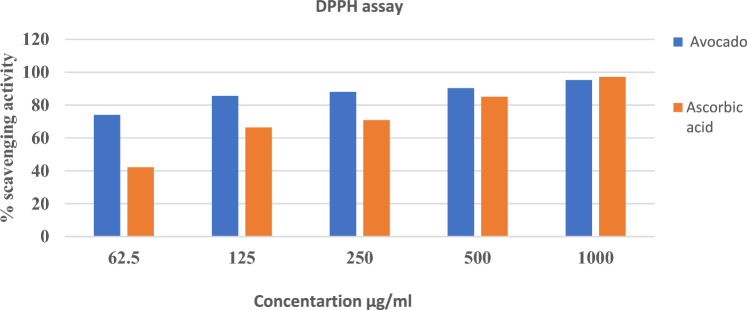
DPPH free radical scavenging activity of TEE of avocado fruit in comparison with ascorbic acid

In the present study, screening of the % radical scavenging activity by DPPH for the TEE of avocado fruit using different extract concentrations (62.5, 125, 250, 500, 1000 µg/ml) revealed that the activity increased in a dose- dependent manner. The % scavenging activity was found to be 74.04%, 85.6%, 88%, 90.29%, and 95.27% respectively, in comparison with the reference standard ascorbic acid 41.02%, 64.47%, 71.65%, 86.08%, and 96.01% respectively, Fig. 1.

The antioxidant activity may be due to the presence of phenolic compounds in the fruit where phenolics are composed of one or more aromatic rings bearing one or more hydroxyl groups and are therefore potentially able to quench free radicals by forming resonance-stabilized phenoxy radicals (Rice-Evans et al. 1996; Bors and Michel 2002).

### In vitro anti-inflammatory activity

In the current study, TEE of avocado exhibited inhibitory activity against COX-1 with IC_50_ of 1.0 ± 0.1 µg/ml as compared to reference standards celecoxib (97.5 ± 0.1 µg/ml) and indomethacin (0.31 ± 0.01 µg/ml). Similarly, TEE inhibited COX-2 enzyme activity with IC_50_ of 0.433 ± 0.058 µg/ml as compared to reference standards celecoxib and indomethacin 0.31 ± 0.01 µg/ml and 0.073 ± 0.006 µg/ml, respectively. Therefore, the avocado extract exhibited anti-inflammatory activity via selective COX-2 inhibition, this may be attributed to compounds belonging to different chemical classes such as unsaturated fatty acids. Termer et al. (2021) reported that the unsaturated fatty acids, linoleic and oleic acids, are responsible for up to 41% of the COX-2 inhibition; in addition to long-chain fatty alcohol persenone A (Kim et al. 2000) along with persin (2-hydroxy-4-oxohenicosa-12,15-dien-1-yl acetate) (Goudarzi et al. 2017) and phenolic acids ferulic acid (Chandel et al. 2018). Moreover, flavonoids such as quercetin, kaempferol and luteolin showed significant inhibition of the COX-2 enzyme (García-Mediavilla et al. 2007; Li et al. 2011).

Cyclooxygenases involved in inflammation exist in two isoforms, COX-1 and COX-2. COX-1, which is present in most tissues, has a beneficial effect maintaining the normal lining of the stomach and intestines by protecting them from digestive juices (Vane and Botting 1998; Hawkey 2001). Contrarily, COX-2 is effectively induced in pathological conditions only such as inflammation, where proinflammtory mediators, cytokines and endotoxins activate its production (Mitchell et al. 1994). Therefore, COX-2 has been considered as the most appropriate target for assessment of anti-inflammatory drugs rather than COX-1, since its inhibition leads to adverse side effects such as reduced mucosal blood flow, retardation of mucous secretion that aids in ulcer healing and decrease in renal blood flow (Wallace and Chin 1997).

### In vivo anti-arthritic activity

#### Acute toxicity studies

There was no mortality at the end of the observation period, hence the LD_50_ was estimated to be larger than 5000 mg/kg and was considered safe. Avocado TEE extract was given at two dose levels (5 and 10%).

#### Effect of daily oral treatment with two dose concentrations of avocado extract on % body weight change

**Fig. 2:**
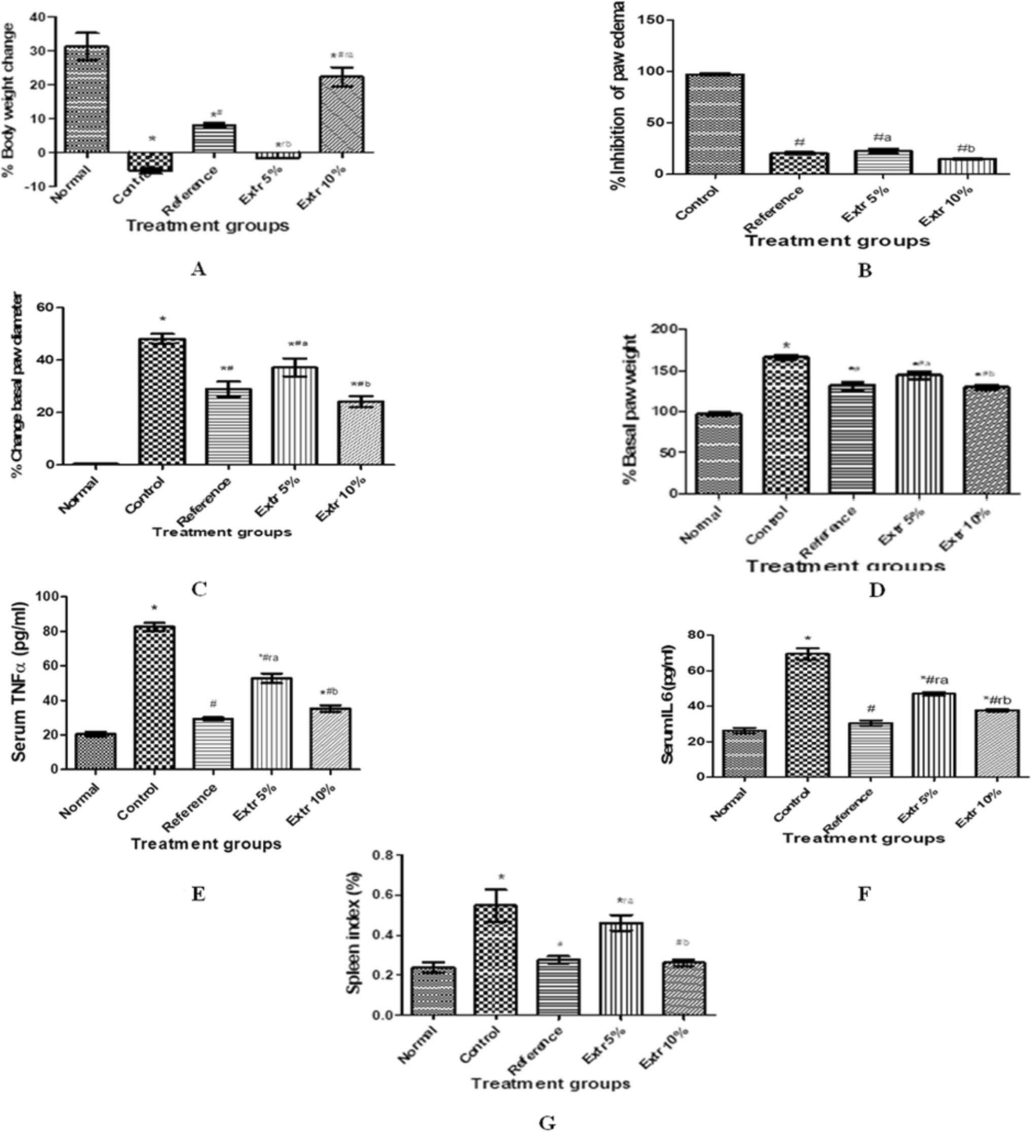
Effect of daily oral treatment with two dose concentrations of avocado extract on % body weight change (**A**) % inhibition of paw edema (**B**), change of basal paw diameter (**C**), and basal paw weight (**D**), serum TNFα (**E**), IL 6 (**F**), and spleen index (**G**). * vs normal; ^#^ vs control; ^r^ vs reference; values not sharing the same alphabet differ significantly at p < 0.05. Each value represents mean ± SEM

At the end of the study period, adjuvant injection induced a significant depression in % body weight changes by 36.6% that of the normal rats. According to Challal et al. (2016), the adjuvant arthritis model is associated with catabolism which manifests in the depression of body weight gain. This was effectively improved by daily oral administration of MTX for 28 days, which significantly increased body weight change by 13.4% that of the diseased group. Likewise, daily oral intake of 10% extract significantly raised body weight change by 27.6%, contrary to the effect of 5% extract, which showed an insignificant change with respect to the control value at p < 0.05 (Fig. 2A).

Avocado fruit extract exerted an increase in the body weight of the experimental animals. This effect could be due to its curative anti-arthritic effect by increasing the absorption of glucose and leucine in rat intestine, which was lowered in arthritic conditions (Somasundaran et al. 1983; Rajendran and Krishnakumar 2010). In addition to its high fat content, the fruit is rich in unsaturated fatty acids (good fat). Polyunsaturated fatty acids are potentially helpful anti-inflammatory agents that might assist individuals at risk in several acute and chronic inflammatory conditions (Calder 2005). Moreover, a human study performed by Monnard and Dulloo (2021) declared that polyunsaturated fats modulate glucose metabolism and alterations in body composition in relation to central adiposity, hence improvement in lean body mass. Therefore, it could be proposed that extract may have improved metabolism and arthritis-induced cachexia (Challal et al. 2016).

#### Effect of daily oral treatment with two dose concentrations of avocado extract on % inhibition of paw edema (volume), change of basal paw diameter and basal paw weight

Adjuvant-injected rats given oral reference drug and extract at two dose levels daily for 28 days exhibited inhibition of paw edema to 20, 22.6 and 15% that of the untreated animals respectively, with superior effect observed in the higher extract concentration (10%) group at p < 0.05 (Fig. 2B). Untreated arthritic animals exhibited a highly significant increase in % change in basal paw diameter to 48% as compared to normal value. Daily oral administration of reference drug induced a significant reduction to 56.3% that of the control diseased group. In a similar manner, higher dose of extract (10%) revealed a significant decrease in % change of basal paw diameter to almost half the value of the control group with an equipotent effect to that of the reference value and a better effect than that of the lower dose extract group at p < 0.05 (Fig. 2C). After excision of the hind limb at the end of the study, adjuvant injection induced a significant elevation of % basal paw weight to 1.7 folds that of the normal value. Daily oral intake of reference and 10% extract for 28 days revealed a marked depression of basal paw weight to almost 78% that of the untreated control group, unlike the weaker effect observed from the low dose (5%) extract group, which showed a significant depression by 13.4% that of arthritic untreated animals at p < 0.05 (Fig. 2D). Results of the current investigation regarding arthritic paws revealed that avocado extract improved paw edema, diameter, and weight due to the presence of bioactive compounds in avocado extract; persenone A, a long chain fatty alcohol (Kim et al. 2000), in addition to persin compound (2-Hydroxy-4-oxohenicosa-12,15-dien-1-yl acetate) (Goudarzi et al. 2017), linoleic and oleic fatty acids (Chandel et al. 2018). Moreover, flavonoids such as quercetin and isorhamnetin have anti-inflammatory and antioxidant activities (Lesjak et al. 2018).

#### Effect of daily oral treatment with two dose concentrations of avocado extract on serum TNF-α

Pro-inflammatory cytokines TNF-α and IL-6 are key mediators in the pathogenesis of rheumatoid arthritis (Sharma et al. 2011; Wang and Zhong 2015) which enhance osteoclastogenesis and expression of matrix metalloproteinases, that culminates in cartilaginous degradation (Thummuri et al. 2015). In the current study***,*** adjuvant injection induced a significant four fold rise in serum TNF-α level with respect to normal value. However, daily oral treatment with reference drug for 28 days normalized serum TNF-α. Oral administration of 5% extract significantly lowered TNF-α to 64% that of the arthritic untreated rats but was still higher than that of the normal and reference values. Likewise, daily intake of 10% extract significantly depressed serum TNF-α to 42.7% that of the untreated group, with an almost equipotent effect to reference standard at p < 0.05 (Fig. 2E).

#### Effect of daily oral treatment with two dose concentrations of the avocado extract on serum IL-6

Similar to TNF-α, untreated arthritic rats showed a significant 2.6-fold elevation in serum IL-6 level. Oral treatment of diseased rats with the reference standard for 28 days significantly reduced levels by 56% with respect to untreated animals. Likewise, daily oral intake of 5 and 10% concentration of the extract significantly decreased serum IL 6 level to 67.6 and 54.4%, respectively as compared to the diseased animal group, however, values remained markedly higher than that of the normal group by 79 and 44%, respectively. Noteworthy, higher extract concentration showed a superior effect to that of the lower dose of extract at p < 0.05 (Fig. 2F). Avocado extract contains compounds such as apigenin (Karunaweera et al. 2015) procyanidins (Cui et al. 2018), monounsaturated fatty acids (Henrotin et al. 1998), chlorogenic and caffeic acids (Meng et al. 2014; Zhang et al. 2014) that could be responsible for reducing the proinflammatory mediator levels of TNF-α and IL-6 in rat serum.

#### Effect of daily oral treatment with two dose concentrations of the avocado extract on spleen index

Splenomegaly in rheumatoid arthritis is attributed to increased β cell proliferation and upregulation of immunoglobulin production (Nishiya et al. 2000; Lazaro and Morel 2015), which is indicated by splenic index. Untreated diseased animals exhibited a significant 2.3-fold increase in spleen index% with respect to that of the normal value. Oral administration of reference drug and 10% extract for 28 consecutive days significantly improved spleen index to near normal value, unlike the 5% extract group, whose spleen index was still significantly high as compared to normal, reference and 10% extract concentration values at p < 0.05 (Fig. 2G). It could be proposed that avocado fruit extract protected spleen from enlargement induced by adjuvant arthritis, showing comparable improvement to MTX**.** This can be explained by the presence of a chemical class identified in avocado fruit for the first time; Lyso-glycerophospholipids that has an immunomodulatory effect. Phosphatidylcholine (PC) supplementation reduced the development of arthritis due to the inhibition of the neutrophil leukocyte-mediated inflammatory reaction (Hartmann et al. 2009).

### Histopathological study

**Fig. 3:**
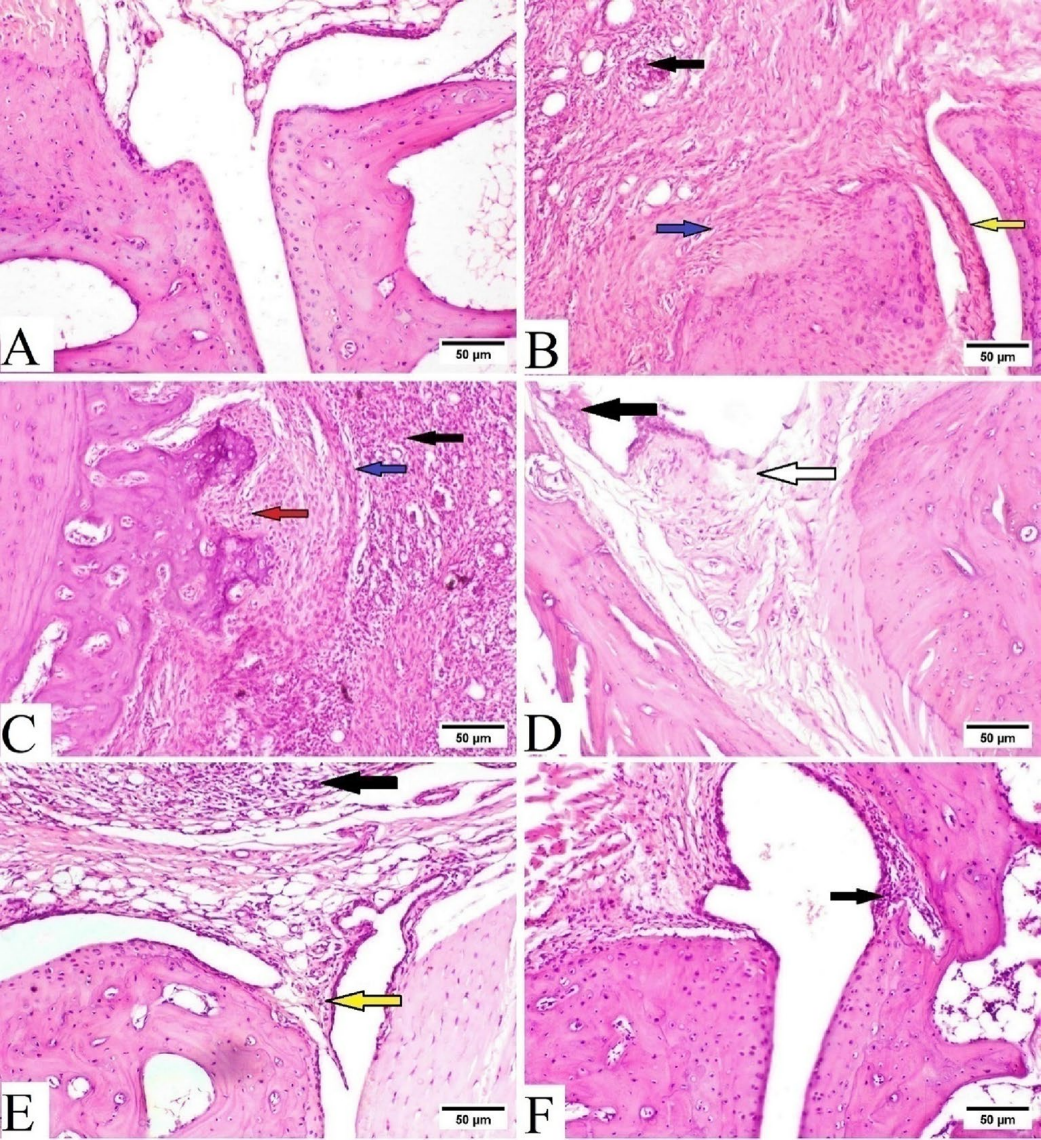
Photomicrographs representative hematoxylin and eosin staining of paw sections of **A** control negative rat showing normal joint, articular surface and synovial membrane. **B** and **C** +ve control; showing massive inflammatory cells infiltration (black arrow), fibroplasia (blue arrow), pannus formation (yellow arrow) and necrosis of cartilage (red arrow). **D** Diseased rat treated with reference standard showing mild inflammatory cells infiltration (black arrow) and edema (white arrow). **E** Diseased rat treated with extract (5%) showing moderate inflammatory cells infiltration (black arrow) and pannus formation (yellow arrow). **F** Diseased rat treated with extract (10%) showing mild inflammatory cells infiltration (black arrow) (H and E stain; scale bar 50 µm)

No histopathological changes were observed in the normal control group; articular surfaces, joint capsules and synovial membranes were intact (Fig. 3A). On the contrary, the +ve control group exhibited serious alterations characterized by severe synovitis, massive inflammatory cell infiltration, marked edema, fibroplasia and pannus formation (Fig. 3 B and C). Additionally, necrosis of cartilage was recorded in some examined sections (Fig. 3C). Meanwhile, treatment with reference induced an improved histological picture, and examined sections exhibited edema, few inflammatory cells infiltration (Fig. 3D) and pannus formation. Likewise, sections from arthritic rats treated with extract 5% showed moderate inflammatory cells infiltration, pannus formation (Fig. 3E), and edema. Furthermore, a more improved histological picture was noticed in sections from arthritic rats treated with extract 10%, as examined sections revealed congested blood vessel and mild inflammatory cells infiltration (Fig. 3F).

### Phytochemical study

#### UPLC/HR-ESI–MS/MS metabolomic profiling of the TEE of avocado fruit

**Fig. 4:**
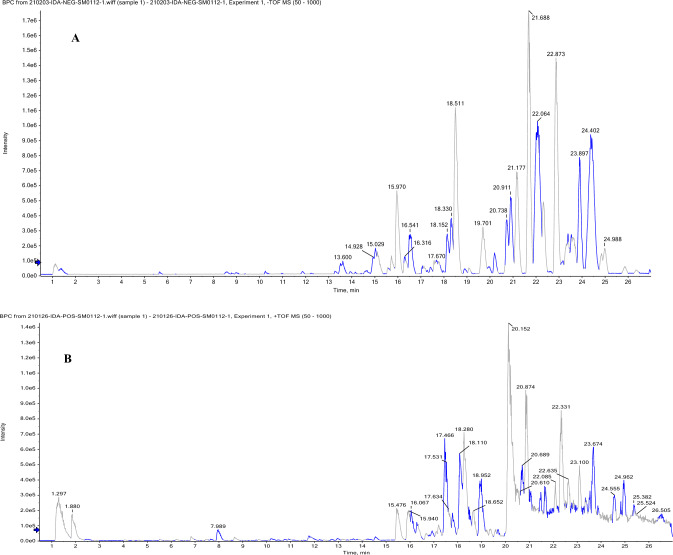
UPLC/ESI–MS chromatograms of TEE of *Persea americana* fruit in negative (**A**) and positive (**B**) ESI–MS modes of ionization

The TEE of *P. americana* fruit was analyzed using reversed-phase UPLC/HR-ESI–MS/MS in negative and positive ionization modes. Throughout the ca. 30 min run time, metabolites were eluted according to their polarity, from the most polar to the least polar. According to available literature data, they were identified using retention time (Rt), MS data (molecular ion, fragmentation pattern, and predicted formula), compared to reported literature data (Di Stefano et al. 2017), MS databases (FooDB, HMDB and Massbank) (Wishart et al. 2018; Horai et al. 2010), in-house library and authentic sources. Figure 4A and B shows representative UPLC chromatograms of selected metabolites. Some of the chemicals discovered had previously been reported in Avocado. The occurrence of 81 metabolites in negative and positive modes belonging to various classes was shown by the high resolution of ESI–MS, including organic acids, phenolic acids, flavonoids, tannins, long chain fatty alcohols, fatty acids, lyso-glcerophospholipids, amino acids and coumarins (Table 1).

**Table 1:** Metabolites tentatively detected via UPLC/HR-ESI–MS/MS analysis of avocado deseeded fruits ethanol extract in both negative and positive ionization modes

Peak no.	R_t_ (min)	Identification	[M−H]^−^	[M+H]^+^	Elemental composition	Error in ppm	MS ions	References
1	1.03	Citric acid	191.0192		C_6_H_7_O_7_^−^	3.1	129, 111	Ragheb et al. (2023)
2	1.06	Malic acid	133.0125		C_4_H_5_O_5_^−^	− 4.5	115, 71	
3	1.16	2-Amino-3-methyl-1-butanol (valinol)	–	104.1066	C_5_H_14_NO^+^	− 3.8	58, 60, 104	Barupal and Fiehn (2019)
4	1.2	Homocitric acid	205.0355	–	C_7_H_9_O_7_^−^	6.1	187, 143, 117, 87	Ragheb et al. (2023)
5	1.24	Perseitol	211.0809	–	C_7_H_15_O^7−^	1.3	193, 157, 121	Yannai (2004)
6	1.40	Protocatechuic acid	153.0194	–	C_7_H_5_O_4_^−^	7.4	109	Figueroa et al. (2018)
7	1.56	Chlorogenic acid	353.0871	–	C_16_H_17_O_9_^−^	1.2	191, 173, 135	Naveed et al. (2018)
8	1.57	Proline	–	116.0710	C_5_H_9_NO_2_^+^	3.0	70	Zhang et al. (2019)
9	1.88	Oxo proline	–	130.0496	C_5_H_9_NO_3_^+^	− 2.5	56, 84, 129	Farid et al. (2022)
10	2.04	Chlorogenic acid isomer	353.0871	–	C_16_H_17_O_9_^−^	1.6	191, 173, 135	Naveed et al. (2018)
11	2.25	Tyrosine	–	182.0810	C_9_H_11_NO_3_^+^	− 1.0	81,119,136, 182	Nishio et al. (2021)
12	2.53	Hydroxy-dimethoxyphenyl-*O*-hexoside	331.1021	–	C_14_H_19_O_9_^−^	0.7	169, 153, 139, 59	HMDB
13	2.69	3,4,5-Trimethoxyphenyl glucoside	345.1172	^−^	C_15_H_21_O_9_^−^	− 2.4	137, 153, 183, 241, 255, 299	Yannai (2004)
14	3.58	Sinapic acid	223.0616	–	C_11_H_11_O_5_^−^	6.9	205, 179, 163, 161	Di Stefano et al. (2017)
15	4.55	Caffeic acid	179.0347	–	C_9_H_7_O_4_^−^	4.4	161, 135	Mata et al. (2016)
16	5.32	Benzyl-O-pentosylhexoside	401.1427	–	C_18_H_25_O_10_^−^	3.8	383, 269, 149, 131	Wei et al. (2021)
17	5.35	Trigonelline	–	138.0548	C_7_H_7_NO_2_^+^	− 1.5	120, 94, 92, 77, 78	Rodrigues and Bragagnolo (2013)
18	5.44	Hydroxy coumarin	–	163.0388	C_9_H_6_O_3_^+^	− 1.0	135, 77	Farid et al. (2022)
19	5.47	Phenylalanine	–	166.0868	C_9_H_11_NO_2_^+^	3.0	73, 77, 103, 120, 131	Farid et al. (2022)
20	5.65	Epi/catechin	289.0712	–	C_15_H_13_O_6_−	1.9	245, 205,152	Widsten et al. (2014)
21	5.63	Abscisic acid	263.1273	–	C_15_H_19_O_4_-	− 1.8	245, 219, 203, 179, 153, 139, 137	Fernández-López et al. (2005)
22	5.70	Quercetin-O-diglucoside	625.1377	–	C_27_H_29_O_17_-	− 3.6	463, 300	Ding et al. (2007)
23	5.77	Quercetin-O-glucuronide	477.0649	–	C_21_H_17_O_13_^−^	− 3.0	301	Vallverdú-Queralt et al. (2015)
24	6.20	Rutin	609.1431	−	C_27_H_29_O_16_^−^	− 3.0	463, 301, 284	Loh and Lim (2018)
25	6.45	Isorhamnetin-O-glucuronide	491.0816	–	C_22_H_19_O_13_^−^	− 0.9	315, 285,271, 257, 256, 255, 243, 162, 151, 113	Allen et al. (2015)
26	6.63	Quercetin-O-pentosylhexoside	595.1273	–	C_26_H_27_O_16_^−^	− 3.5	463, 300	Akan (2021)
27	6.69	Kaempferol-O-pentosylhexoside	579.1315	–	C_26_H_27_O_15_^−^	5.1	447, 285, 151	Akan (2021)
28	6.74	Quercetin-O-hexoside	463.0877	–	C_21_H_19_O_12_^−^	1.2	300, 271, 179, 151	Mosić et al. (2019)
29	6.96	Acteoside	623.19585	–	C_29_H_35_O_15_^−^	− 1.9	477, 461, 179, 161, 153, 135	Tao et al. (2018)
30	7.06	1-Palmitoleoyl-2-hydroxy-sn-glycero-3-phosphoethanolamine	–	452.2779	C_21_H_42_NO_7_P^+^	1.5	434, 311, 295, 237	Ragheb et al. (2023)
31	7.11	Vanillic acid	167.03429	–	C_8_H_7_O_4_^−^	2.4	152, 123, 108	Horai et al. (2010)
32	7.21	Scopoletin	191.0341	–	C_10_H_7_O_4_^−^	1.1	176, 148, 120, 108	Farid et al. (2022)
33	7.25	Luteolin-*O*-glucoside	447.09445	–	C_21_H_19_O_11_^−^	5.1	284, 241, 133	Brito et al (2014)
34	7.30	6-hydroxynon-7-enoic acid	171.1013		C_9_H_15_O_3_	− 1.5	125, 127	Farid et al. (2022)
35	7.42	Quercetin-O-pentosylrhamnoside	579.13258	–	C_26_H_27_O_15_-	− 3.2	300	Liu et al. (2016)
36	7.53	Quercetin-*O*-rhamnoside	447.09149	–	C_21_H_19_O_11_^−^	− 1.6	301, 284, 273, 179	Rojas-Garbanzo et al. (2017)
37	7.57	Isorhamnetin-*O*-hexoside	477.0995	–	C_22_H_21_O_12_^−^	6.7	314	Chen et al. (2015)
38	7.62	Kaempferol-*O*-hexosylrhamnoside	593.14958	–	C_27_H_29_O_15_^−^	− 0.9	447,431, 285, 151	Figueroa et al. (2018)
39	8.16	Hydroxytyrosol 1-*O*-glucoside	–	317.1238	C_14_H_20_O_8_^+^	2.2	299,281,147,137	Peralbo-Molina et al. (2012)
40	8.26	Kaempferol-*O*-rhamnoside	431.0962	–	C_21_H_19_O_10_^−^	− 2.5	285, 257, 229	Ding et al. (2007)
41	8.26	Ferulic acid	193.0503	–	C_10_H_9_O_4_^−^	3.8	179, 161, 135	Santana et al. (2019)
42	9.48	Hydroxy-oxo hexadecanoic acid	285.20684	–	C_16_H_29_O_4_^−^	2.8	267, 239, 199, 125, 59	Allen et al. (2015)
43	9.71	Epi-dihydrophaseic acid	281.13794	–	C_15_H_21_O_5_^−^	1.5	263, 253, 237, 221, 219, 153, 113	Wang et al. (2021)
44	9.75	Quercetin	301.03609	–	C_15_H_9_O_7_-	2.5	255, 179, 151	Rojas-Garbanzo et al. (2017)
45	10.20	Naringenin	271.05968	–	C_15_H_11_O_5_^−^	− 1.5	227, 177, 151, 119	Fabre et al. (2001)
46	10.58	O,O-Dimethyl caffeic acid	207.06587	–	C_11_H_11_O_4_^−^	3.3	193, 179, 161, 135, 121	Yannai (2004)
47	10.88	Luteolin	285.0410	–	C_15_H_9_O_6_^−^	5.8	267, 151, 133	Yang et al. (2021)
48	11.43	Procyanidin dimer B1/B2	577.13309	–	C_30_H_25_O_12_^−^	− 1.7	451, 425, 289	Rockenbach et al. (2012)
49	11.89	Oxohexadecenoic acid	267.19561	–	C_16_H_27_O_3_^−^	0.5	113, 87, 59	Wang et al. (2021)
50	12.36	Oxohexadecanoic acid	269.2122	–	C_16_H_29_O^3−^	4.0	251, 225, 169, 149	Wang et al. (2021)
51	13.04	Avocadynone acetate	323.22316	–	C_19_H_31_O_4_^−^	4.6	305, 263, 233, 171	Wang et al. (2021)
52	13.86	1-Heptadecanoyl-glycero-3-phosphocholine LGPC (17:0)	–	510.3593	C_25_H_52_NO_7_P^+^	7.7	184	Ragheb et al. (2023)
53	14.48	Avocadyne acetate	325.23591		C_19_H_33_O_4_^−^	4.4	265, 247, 207	Wu et al. (2019)
54	14.79	Palmitamide	–	256.1911	C_16_H_33_NO^+^	1.5	239, 89, 69, 71	Watson (2006)
55	14.8	Dihydroxy-octadecadienoate	311.1695	–	C_18_H_31_O_4_^−^	1.8	293, 275, 265, 249	Figueroa et al. (2018)
56	15.02	Hydroxy hexadecanoic acid	271.22725	–	C_16_H_31_O_3_-	1.8	227, 211	Deas et al. (1974)
57	15.91	Avocadyne 16-Heptadecyne-1,2,4-triol	283.2277	−	C_17_H_31_O_3_−	3.1	265, 247, 237, 235, 221, 207, 59	Wu et al. (2019)
58	15.91	Lysophosphatidylcholine(18:3)) 6,12,15	–	518.3238	C_26_H_48_NO_7_P^+^	− 0.6	184, 124, 104	Ragheb et al. (2023)
59	16.54	Avocadene acetate	327.25383	–	C_19_H_35_O_4_^−^	2.6	309, 285, 265, 249, 73	Wu et al. (2019)
60	16.76	Lysophosphatidylcholine(16:1(9Z)/0:0 1-Palmitoleoyl-glycero-3-phosphocholine	–	494.3230	C_24_H_48_NO_7_P^+^	− 2.3	– 476, 311, 184, 104	Ragheb et al. (2023)
61	17.55	LysoPC(18:2(9Z,12Z)/0:0) 2-Linoleoyllysophosphatidylcholine (18:2)	–	520.3378	C_26_H_50_NO_7_P^+^	− 3.7	– 502, 184, 104	
62	18.51	Avocadene = 1,2,4-trihydroxy-16-heptadecene	285.2418	–	C_17_H_33_O_3_^−^	1.6	267, 249, 223, 207, 209, 73	Ding et al. (2007)
63	18.62	Lysophosphatidylcholine(18:1(9Z)/0:0) Oleoyl-sn-glycero-3-phosphocholine; LGPC (18:1)	−	522.3549	C_26_H_52_NO_7_P^+^	− 0.9	184, 86	Ragheb et al. (2023)
64	18.90	Oleoyl-sn-glycero-3-phosphocholine; LGPC (18:1) isomer	–	522.3535	C_26_H_52_NO_7_P^+^	− 3.7	504, 339, 184	
65	20.16	Heptadecadienoic acid	265.2163		C_17_H_29_O_2_^−^	0.4	247, 165	HMDB database
66	20.39	2-Linoleoylglycerol Glyceryl-1-monolinoleate	–	355.2849	C_21_H_38_O_4_^+^	1.6	355, 337, 263, 245, 95, 81, 69	Ragheb et al. (2023)
67	20.67	Hexadecenoic acid	253.21711	–	C_16_H_29_O_2_^−^	3.6	235, 207	Smith et al. (2019)
68	21.16	Hydroxynonadecanoic acid	313.2743	–	C_19_H_37_O_3_^−^	1.8	277, 265, 251, 237	Deas et al. (1974)
69	21.54	Hexadecanoic acid	255.2327	–	C_16_H_31_O_2_^−^	3.3	237	Yang et al. (2015)
70	21.74	2-Hydroxysterculic acid	309.2428	–	C_19_H_33_O_3_^−^	1.1	291, 265	Watson (2006)
71	22.36	N-Oleoylethanolamine	–	326.3737	C_20_H_39_NO_2_^+^	− 5.2	309, 135, 62	Hofmann et al. (2003)
72	22.57	Persenone A	377.27085	–	C_23_H_37_O_4_^−^	5.9	359, 335, 287, 57	Kim et al. (2000)
73	22.83	Trihydroxy-nonadecane	315.28851	2−	C_19_H_39_O_3_^−^	− 2.7	297, 279, 267, 261, 255, 239, 237, 57	Allen et al. (2015)
74	23.31	Persin	379.28166	–	C_23_H_39_O_4_^−^	6.9	319, 305, 289	Oelrichs et al. (1995)
75	23.54	1-Docosadienoyl-glycero-3-phosphocholine)	–	576.3995	C_30_H_58_NO_7_P^+^	5	518, 184	Ragheb et al. (2023)
76	24.49	Linolenic acid	277.2152	–	C_18_H_29_O_2_^−^	− 3.6	277, 259, 233, 59, 41	Cowan and Wolstenholme (2016)
77	24.99	Heptadecanoic acid	269.2478		C_17_H_33_O_2_^−^	1.1	251, 225, 197, 141	Allen et al. (2015)
78	25.59	Oleic acid	281.2475	–	C_18_H_33_O^−^	0.1	181	Farag et al. (2015)
79	24.73	9-Methyloctadecanoic acid	–	299.3534	C_19_H_38_O_2_^+^	2	299,281, 263, 109, 97, 81, 69	Okparauka et al. (2019)
80	25.64	Octadecanamide	–	284.2946	C18H37NO^+^	− 0.5	57, 60, 88	Castillo-Peinado et al. (2019)
81	26.44	20-Alpha-dihydroprogesterone	–	317.2473	C_21_H_32_O_2_^+^	− 0.6	299, 271, 159, 145, 131, 91	Rovensky et al. (2004)

#### Organic acids

Three organic acids were identified in peaks 1, 2, and 4. Citric acid at [M−H]^−^ 191 (C_6_H_7_O_7_)^−^, malic acid at [M−H]^−^ 133(C_4_H_5_O_5_)^−^ and homocitric acid at [M−H]^−^ 205(C_10_H_17_O_4_)^−^ (Ragheb et al. 2023). While citric acid gave a fragment at *m/z* 111 corresponding to [M−H–CO_2_–2H_2_O]^−^. Organic acids have been shown to have numerous biological functions such as antioxidant and anti-inflammatory activities. Both citric acid and l-malic acid significantly decreased inflammatory cytokine TNF*-α* activity and inhibited ADP-induced platelet aggregation (Tang et al. 2013).

#### Phenolic acids

Seven phenolic acids were identified in this study. Phenolic acids yielded deprotonated [M−H]^−^ fragment ion in negative ion mode UPLC/MS which give characteristic diagnostic fragments in MS/MS resulting from either dehydration (–H_2_O), decarboxylation(–COO), demethylation (–CH_2_–) or demethoxylation –OCH_2_^−^, e.g. Protocatechuic acid (peak 6) [M−H]^−^ 153 (C_7_H_5_O_4_)^−^ (Figueroa et al. 2018); Chlorogenic acid and its isomer (peaks 7, 10) [M−H]^−^ 353 (C_16_H_17_O_9_)^−^ (Naveed et al. 2018); Sinapic acid (peak 14) [M−H]^−^ 223 (C_11_H_11_O_5_^**−**^) (Di Stefano et al. 2017); Caffeic acid (peak 15) [M−H]^−^ 179 (C_9_H_7_O_4_^−^) (Mata et al. 2016);*O*,*O*-Dimethyl caffeic acid (peak 46) [M − H]^−^ 207 (C_11_H_11_O_4_^−^) (Yannai 2004),the fragments 193 and 179 correspond to loss of dimethyl (C_2_H_4_) yielding caffeic acid (C_9_H_7_O_4_^−^). Vanillic acid (peak 3), *m*/*z* 167 [M−H]^−^ (C_8_H_7_O_4_)^−^ (Horai et al. 2010) and Ferulic acid (peak 41) *m*/*z* 193 [M−H]^−^ (C_10_H_9_O_4_)^**−**^** (**Santana et al. 2019). Phenolic acids have a wide array of biological activities including antioxidant and anti-inflammatory activities (Song et al. 2020; Ullah et al. 2020). They prevent the formation of reactive oxygen species (ROS), which cause cell damages and are associated with the development of a variety of disorders. (Damasceno et al. 2017). Additionally, chlorogenic acid showed antioxidant and anti-inflammatory activities in vitro and in vivo studies (Wang et al. 2022).

Acteoside (peak 29) (Tao et al. 2018) is a cinnamate ester with disaccharide residue (rhamnosyl-(1- > 3)-beta-d-glucoside) *m/z* 623.19585 [M−H]^−^ C_29_H_35_O_15_. It is a phenylpropanoid glycoside that demonstrated various pharmacological activities, including antioxidant, anti-inflammatory, bone and cartilage protection in addition to other health benefits (Xiao et al. 2022).

#### Flavonoids

Flavonoids undergo a fragmentation pathway within aglycone through the Retro-Diels–Alder (RDA) reaction yielding small neutral molecules and fragments, e.g., CO_2_, CO and H_2_O (Cuyckens and Claeys 2004). Fourteen different flavonol glucosides were characterized in the studied extract (quercetin, isorhamnetin, kaempferol, luteolin). Eight different quercetin derivatives were detected. In accordance with an *O*-glycosidic cleavage: Quercetin-*O*-glucoside (peak 22) at [M−H]^−^ 625(C_27_H_29_O_17_^−^) (Ding et al. 2007). Quercetin-*O*-glucuronide (peak 23) [M−H]^−^ 477 (C_21_H_17_O_13_^−^) with fragments *m/z* 301[M−H]^−^-176] (Vallverdú-Queralt et al. 2015); Rutin (peak 24) [M−H]^−^ 609 (C_27_H_29_O_16_^−^) with fragments *m/z* 463 [M−H–146 rhamnose]^−^ and *m/z*301[M−H–rhamnose–hexose–]^−^ (Loh and Lim 2018); Quercetin-*O*-pentosyl hexoside (peak 26) 595 [M−H]^−^ (C_26_H_27_O_16_)^−^, *m/z* 463 [M−H–132 pentose]^−^ and *m/z* 301 [M−H–pentose-hexose–]^−^ (Akan 2021); Quercetin-*O*-hexoside (peak 28) [M−H]^−^ 463 (C_21_H_19_O_12_^−^),Quercetin-O-pentosylrhamnoside (peak 35) [M−H]^−^ 579 (C_26_H_27_O_15_^−^) (Mosić et al. 2019). Quercetin-*O*-rhamnoside (peak 36) [M−H]^−^ 447 and quercetin (peak 44) *m*/*z* 301 [M−H]^−^ (Rojas-Garbanzo et al. 2017). It was reported that quercetin-*O*-rhamnoside has beneficial effects on the swift healing of skin injuries (Elloumi et al. 2022). In addition, quercetin acts as an antioxidant, anti-inflammatory and immune modulator (Anand David et al. 2016; Massi et al. 2017). Three Kaempferol derivatives were detected showing the characteristic fragment ions at *m/z* 285 [aglycone–H] ^–^and 284 [aglycone–2H]^−^. Two kaempferol diglycosides and one monoglycoside were detected corresponding to peaks 27, 38 and 40: Kaempferol-*O*-pentosylhexoside (peak 27) [M−H]^−^ 579 (C_26_H_27_O_15_^−^), *m/z* 447 [M−H–132]^−^ corresponding to loss of pentosyl group and *m/z* 285, 151 represent aglycone (Akan 2021) [M−H–pentose 132-hexose 162]^−^; Kaempferol-*O*-hexosylrhamnoside; (peak 38) [M−H]^−^ 593 (C_27_H_29_O_15_^−^). Moreover, (peak 40) was identified as Kaempferol-*O*-rhamnoside [M−H]^−^ 431 (C_21_H_19_O_10_^−^) (Ding et al. 2007). Kaempferol has a potent antioxidant activity (Deng et al. 2019), which can be found in a wide variety of herbs and plant families with anti-inflammatory activity (Periferakis et al. 2022).

Luteolin derivatives were detected as luteolin-*O*-glucoside (peak 33) *m/z* 447[M−H]^−^, *m/z* 284 [M-2H-162]^−^ corresponding to luteolin aglycone (Brito et al. 2014). Additionally, luteolin aglycone (peak 47) at *m/z* 285[M−H]^−^ was detected (Yang et al. 2021). Previous research has revealed that luteolin is an anti-inflammatory and anti-oxidative agent, inhibiting the formation of prostaglandin E2 and nitric oxide (NO), as well as the expression of their related enzymes. (Park and Song 2013). Moreover, two isorhamnetin glycosides were detected; isorhamnetin-*O*-glucuronide (peak 25) at [M−H]^−^ 491 (C_22_H_19_O_13_^−^) (Allen et al. 2015) and isorhamnetin-*O*-hexoside (peak 37) at [M−H]^−^ 477(C_22_H_21_O_12_^−^) (Chen et al.^2015^). In addition, the presence of a hexose in the molecule was confirmed by the loss of a mass of 162. Naringenin (peak 45) [M−H]^−^ 271 (C_15_H_11_O_5_^−^) yielded major fragments ion at *m*/*z* 227, 177, 151 and 119 (Fabre et al. 2001).

#### Tannins

Epicatechin (peak 20) *m*/*z* 289 [M−H]^−^ (C_15_H_13_O_6_)^**–**^ has been previously reported in avocado peel (Calderón-Oliver et al. 2016; Wong et al. 2016). Procyanidin dimer B1/B2 (peak 48) *m*/*z* 577 [M−H]^−^ (C_30_H_25_O_12_)^−^ yielded major fragment ions at *m*/*z* 451(heterocyclic ring fusion), *m*/*z* 425 and *m*/*z* 289, the latter correspond to catechin moiety(Rockenbach et al. 2012).

#### Long-chain fatty alcohols

Avocado fruit is reported to be enriched with straight-chain fatty alcohols with different degrees of unsaturation, alkyl chain length, hydroxylation, and subsequent acetylation (Bhuyan et al. 2019). These fatty alcohols are reported to exhibit antiviral, cytotoxic, antifungal, antioxidant and antimicrobial activities (Mukherjee et al. 2013). In our study, long-chain polyhydroxylated fatty alcohols were detected in avocado fruit crude extract in peaks 51, 53, 57, 59, 62, 73, 70, 72, and 74. A common fragmentation pattern was observed in which the deprotonated molecular ion [M−H]^−^ undergoes dehydration [M–H–H_2_O]^−^, decarbonylation [M−H–CO]^−^, decarboxylation [M−H–COO]^−^, demethoxylation [M−H–OCH_2_]^−^ and deacetylation [M−H–COCH_2_]^−^_._ In peaks 51, 53 and 59 fatty alcohols were esterified with acetic acid as manifested with loss of acetyl moiety (42 amu, CH_2_CO^−^) and identified as avocadynone acetate (peak 51) (Wang et al. 2021) at [M−H]^−^ 323 C_19_H_31_O_4_^−^, avocadyne acetate (peak 53) (Wu et al. 2019) which showed [M−H]^−^ 325 C_19_H_33_O_4_^−^ and avocadene acetate at [M−H]^−^ 327 C_19_H_35_O_4_^−^, respectively. Where avocadynone acetate showed main product ions at *m/z* 305 [ M-H-H_2_O]^−^, *m/z* 263 [M-H-COCH_2_ acetyl group]^−^ and *m/z* 233 [M−H–OCH_2_ methoxy group].The other fatty alcohol identified at *m/z* 283, 285, 377, 315, and 379 as avocadyne (16-Heptadeyne-1,2,4-triol) (peak 57) C_17_H_31_O_3_^−^ (Wu et al. 2019); avocadene (1,2,4-trihydroxy-16-heptadecene) (peak 62) C_17_H_33_O_3_^−^ (Ding et al. 2007); persenone A (peak 72) C_23_H_37_O_4_^−^ (Kim et al. 2000); Trihydroxy-nonadecane (peak 73) C_19_H_39_O_3_^−^ (Allen et al. 2015); persin (peak 74) C_23_H_39_O_4_^−^ (Oelrichs et al. 1995). Persenone A is a promising agent that prevent inflammation-associated diseases (Kim et al. 2000). Persenone A along with persin were found to suppress superoxide (O_2_^−^) and nitric oxide (NO) generation in cell culture (Domergue et al. 2000; Hashimura et al. 2001).

Perseitol (peak 5) as sugar alcohol *m/z* 211 [M−H]^−^, C_7_H_15_O_7_^−^, MS/MS fragment ion at *m/z* 101 (Yannai 2004).

#### Long-chain fatty acids

Lauraceous acetogenins are a family of long-chain fatty acids with biologically active derivatives (Rodriguez-Saona and Trumble 2000). In this study, eleven long-chain fatty acids were detected in avocado fruit crude extract; 2 oxo-fatty acids (peaks 49 and 50) were detected and identified as oxohexadecenoic acid at [M−H]^−^ *m/z* 267, C_16_H_27_O_3_^−^ and oxohexadecanoic acid at [M−H]^−^*m/z* 269, C_16_H_29_O_3_^−^, they are detected for the first time in avocado fruit. It produced the MS2 fragment at *m/z* 113, 87 and 59. The main product ions *m/z* 251 due to loss of H_2_O^−^, *m/z* 225 (Wang et al. 2021). Hydroxy-fatty acids derivatives (peaks 42, 55, 56 and 68) were detected as Hydroxy-oxo hexadecanoic acid (peak 42) at [M−H]^−^ *m/z* 285, C_16_H_29_O_4_^–^ (Allen et al. 2015) produced fragments ion at *m/z* 267 due to loss of (–H_2_O), at *m/z* 239 due to loss of carbonyl group, at *m/z* 199, 125 and 59. Dihydroxy-octadecadienoate (peak 55) at [M−H]^−^ *m/z* 311, (C_18_H_31_O_4_)^−^ (Figueroa et al. 2018) and hydroxy hexadecanoic acid (peak 56) at [ M–H]^−^ *m/z* 271, C_16_H_31_O_3_^−^ (Deas et al. 1974) produced fragment ions *m/z* 227 due to decarboxylation (–COO^−^) and *m/z* 211 due to loss of –H_2_O^−^ and hydroxynonadecanoic acid (peak 68) at [M−H]^−^ 313, C_19_H_37_O_3_ (Deas et al. 1974). Moreover, 6 straight-long chain, fatty acids in peaks (65, 67, 69, 76, 77, and 78) were detected. Four of them are long unsaturated fatty acids; Heptadecadienoic  acid (peak 65) (HMDB database) was detected at [M−H]^−^ *m/z* 265 C_17_H_29_O_2_^−^, produced fragments ions *m/z* 247 and 165, Hexadecenoic acid (peak 67) at [M−H]^–^ *m/z* 253 C_16_H_29_O_2_^−^ (Smith et al. 2019), linolenic acid (peak 76) was noticed at *m/z* 277 [M−H]^−^ (Cowan and Wolstenholme 2016) and oleic acid was observed at *m/z* 281 [M−H]^−^ (peak 78) (Farag et al. 2015), in addition to two saturated fatty acids detected (peaks 69 and 77); hexadecanoic acid (Yang et al. 2015) and heptadecanoic acid (Allen et al. 2015) were detected at [M−H]^−^ *m/z* 255 and 269, respectively. Heptadecanoic acid produced fragments ions *m/z* 251 due to loss of H_2_O, 225 due to carboxylic group (–COO–), 197 carbonyl group (–CO–), and 141. Moreover, 9-Methyl octadecanoic acid (peak 79) (Okparauka et al. 2019). Palmitamide, is a fatty acid amide (peak 54) [M+H]^+^ 256 C_16_H_33_NO^+^, it yielded ions *m/z* 239, 89, 71; N-Oleoylethanolamine (ethanol amide of oleic acid) (peak 71), [M+H]^+^ 326, C_20_H_39_NO_2_^+^ (Hofmann et al. 2003) (HMDB), and octadecanamide (peak 80) [M+H]^+^ protonated ion 284 C_18_H_37_NO^+^ (Castillo-Peinado et al. 2019). In addition to 2-linoleoyl glycerol peak (66). (9Z,12Z-octadecadienoic acid, 2-glyceryl ester) (C_21_H_38_O_4_^+^) that was detected in the positive mode with protonated ions [M+H]^+^ 355 (Ragheb et al. 2023). It was reported that PUFA (polyunsaturated fatty acids) are useful in alleviating several diseases such as cardiovascular, inflammatory heart diseases, atherosclerosis, autoimmune disorder and diabetes (Finley and Shahidi 2001). Siswadi and Saragih (2021) reported that hexadecanoic acid possesses important biological activities such as antioxidant, antimicrobial, hypocholesterolemic and anti-inflammatory activities. In metabolic illness mouse models, hexadecenoic acid has anti-inflammatory effects on hepatic steatosis and insulin signaling. It has recently been described in human circulating monocytes and monocyte-derived macrophages as anti-inflammatory (Astudillo et al. 2018). Linolenic acid was shown to block the synthesis of prostaglandin which results in reduction of inflammation and prevention of certain chronic diseases. It was observed that the fatty acids and their amides can cover a wide range of therapeutic indications such as bacterial infections and inflammations (Tanvir et al. 2018).

#### Lyso glycerophospholipids (LGPLs)

The molecular structure of LGPLs consists of a hydrophobic acyl chain(s) attached to the positions 1 and 2 of the glycerol backbone and in position 3 hydrophilic phosphate head group was attached. Various kinds of LGPLs were discovered based on the type of hydrophilic head. LGPLs are structural components cell membranes that can function as signing molecules in a variety of physiological and biological aspects (Suárez-García et al. 2017). In this study, there were two classes of Lyso-glycerophospholipids; Lyso-glycerophosphoethanolamines and Lyso-glycerophosphocholines, including 8 metabolites, were detected for the first time in the fruit under study and eluted in the second half part of the chromatogram, they were mainly detected in the positive ion mode, as the phosphate group accepts a proton during MS analysis. They were: Lyso-glycerophosphoethanolamines; LGPEs 1-palmitoleoyl-2-hydroxy-sn-glycero phosphoethanolamine (peak 30); Lysogycerophosphocholines; LGPCs compounds as1-Heptadecanoyglycero-3-phosphocholine LGPC (peak 52); Lyso-phosphatidylcholine (peak58); 1-Palmitoleoylglycerophosphocholine(peak60); 2-Linoleolylsophosphatidylcholine (peak 61); Oleoyl-sn-glycero-3-phosphocholine; LGPC(peak63). Oleoyl-sn-glycero-3-phosphocholine; LGPC isomer (peak 64) and 1-Docosadienoyl-glycero-3-phosphocholine (peak 75) (Ragheb et al. 2023).

The detected LGPCs and LGPEs were identified with molecular formula CxHxNO8P, where the glycerol moiety acylated with one or two fatty acid(s), the phosphate group at position 3 was either connected to choline (phosphatidylcholine) or ethanolamine (phosphatidylethanolamine). The product ion at *m/z* 184 is a characteristic fragment ion to the phosphocholine head group. (Murphy and Axelsen 2011). Glycerophospholipids (GPLs)were also reported to decrease the pro-inflammatory cytokines as IL-1β, TNF-α and NO in LPS-activated microglia (Kim et al. 2020). Additionally, GPLs reduced the gastric mucosal lesions in rats after treatment with NSAIDs (Küllenberg et al. 2012).

#### Amino acids

Avocado contains 18 of the 22 amino acids required for consumption including all the eight essential amino acids. It contains more protein than an equivalent amount in cow’s milk, which makes them a high-quality protein food with special value to vegetarian diet (Caballero et al. 2005). In this study, four amino acids were identified (peaks 8, 9, 11, and 19), proline (peak 8) [M+H]^+^ 116 (C_5_H_9_NO_2_^+^). The fragmentation reaction of protonated proline was simple. Only one fragment at *m*/*z* 70 was observed, which was assigned as [M + H − H_2_O − CO]^+^ (Zhang et al. 2019); oxyproline (peak 9) [M+H]^+^ 130 (C_5_H_9_NO_3_^+^) (Farid et al. 2022); tyrosine (peak 11) protonated ions [M+H]^+^ 182 (C_9_H_11_NO_3_^+^), the loss of H_2_O + CO from protonated tyrosine led to the formation of a fragment ion at *m/z* 136, Further loss of NH_3_ resulted in the formation of the fragment ion at *m/z* 119 (Nishio et al. 2021); phenylalanine (peak19) [M+H]^+^ 166 (C_9_H_11_NO_2_^+^) the major fragmentation pathway of protonated phenylalanine started from the loss of H_2_O + CO to form a fragment ion at *m/z* 120. A fragment ion at *m/z* 103 was formed by the further loss of NH_3_ (Farid et al. 2022). Muscle weakness, poor wound healing, weakened immunity, stunted growth, and dull skin and hair are all effects of deficiency of essential amino acids. Avocados are one of the few fruits that contain all essential amino acid (Caballero et al. 2005).

#### Coumarins

Two coumarins were identified in our study in the positive and negative modes (peaks 18, 32)**;** hydroxy coumarin (peak18) protonated ion [M+H]^+^163 (C_9_H_6_O_3_^+^) showed fragment ions at *m/z* 135 due to loss of CO from protonated hydroxyl coumarin and *m/z* 77 corresponding to C_5_HO^+^; scopoletin (peak 32) [M−H]^−^ 191 (C_10_H_7_O_4_)^−^ (Farid et al 2022), showed fragmentation ions *m/z*176 [M–H–H_2_0]^−^ due to dehydration *m/z* 148 [M–H–H_2_0–OCH_2_]^−^ *m/z* 120 corresponding to (C_8_H_7_O) due to (demethylation—CH_2_). It was reported that scopoletin has antiarthritic activity, significantly reducing the production of IL-6 in fibroblast-like synoviocytes (FLS) in arthritic rats (Dou et al. 2013). Moreover, hydroxy coumarin and its derivatives showed important pharmacological effects, as analgesic, anti-arthritic and anti-inflammatory effects (Adami et al.^1959^; Chiarino et al. 1988; Luchini, et al. 2008; Jung and Park 2009).

#### Miscellaneous

Hydroxy-dimethoxyphenyl-O-hexoside (peak 12) [M−H]^−^ 331 (C_14_H_19_O_9_^−^) showing fragments *m/z* 169,153 (HMDB database). 3,4,5-Trimethoxyphenyl glucosid( peak13) (Yannai 2004) (HMDB) [M−H]^−^ C_15_H_21_O_9_^−^ 345; Epi-Dihydrophaseic acid (peak43) [M−H]^−^ 281 (C_15_H_21_O_5_)^−^, showing fragments *m/z* 263 [M−H–H_2_O–]^−^, *m/z* 253 [M−H–CO]^−^ and *m/z* 237 [M–H–COOH]^−^ (Wang et al. 2021); Benzyl-O-pentosyl-hexoside (peak16) [M–H^−^] 401 (C_18_H_25_O_10_^−^), *m/z* 383 and 269 (Wei et al. 2021); Hydroxytyrosol 1-*O*-glucoside peak (39) protonated ions [M+H]^+^ 317 (C_14_H_20_O_8_^+^) (C_14_H_20_O_8_^+^) yielded fragment ions *m/z*299 corresponding to (C_14_H_19_O_7_^+^) due to dehydration [M + H–18]^+^ (loss of H_2_O), *m/z* 281 and *m/z* 137 (Peralbo-Molina et al. 2012). It was reported that Hydroxy Tyrosol has high antioxidant efficiency due to the presence of the *O-dihydroxy phenyl* moiety (Karković Marković et al. 2019)*.* Trigonelline (alkaloid) (peak 17) [M+H]^+^ protonated ion 138 (C_7_H_7_NO_2_^+^) (Farid et al. 2022; Rodrigues and Bragagnolo 2013).

Abscisic acid is a sesquiterpene (peak 21) [M–H]^−^ 263 (C_15_H_19_O_4_^−^) (Fernández-López et al. 2005); 2-Amino-3-methyl-1-butanol (Valinol, amino alcohol) (peak3) (Barupal and Fiehn 2019; HMDB database) (C_5_H_14_NO^+^) protonated ions [M+H]^+^ 104 yielded fragment ions *m/z*60 corresponding to C_2_H_6_NO^+^. It was reported that valinol can act as non-opioid analgesic agents (Fuchs et al. 2014). Moreover, 20-alpha-dihydroprogesterone was also identified at (peak 81) protonated ion [M+H]^+^ 317 (C_21_H_32_O_2_^+^) (Rovensky et al. 2004; HMDB database) yielded fragment ions *m/z*299 due to loss of H_2_O(dehydration), and *m/z* 271 due to loss of carbonyl.

## Conclusions

Avocados have become an increasingly popular food in recent years as they are rich in antioxidant content. Regular consumption of avocado fruits might improve antioxidant defenses of the body and could be considered an excellent source of anti-inflammatory compounds.

Screening of the in vitro antioxidant and anti-inflammatory activities of TEE of *P. americana* fruit showed a high antioxidant activity due to the presence phenolics, flavonoids, fatty alcohols and fatty acids. Moreover, the TEE of the fruit exerted a significant in vivo anti-arthritic activity by improving arthritic paw parameters of the diseased experimental animals and serum inflammatory biomarkers (IL-6 and TNF-α). This was further emphasized by the histopathological study of the rats' excised hindlimbs, which showed milder inflammatory cell infiltration and improvement in histological picture. A study of the chemical constituents of the TEE of the fruit by UPLC/HR-ESI–MS/MS spectrometry led to the identification of 81 metabolites belonging to various classes, most of which possess antioxidant and anti-inflammatory activities.

This study suggests recommending avocado fruit consumption by patients who are prone to arthritic problems. According to the findings of the current investigation and a review of traditional medicine literature, avocado fruit extract could be considered as a promising antioxidant and anti-arthritic natural product.

## Data Availability

The data that support the finding of the study are available from the corresponding author (Dina Atef Waly) upon reasonable request.
